# The DNA repair kinase ATM regulates CD13 expression and cell migration

**DOI:** 10.3389/fcell.2024.1359105

**Published:** 2024-06-12

**Authors:** Louise K. Stevenson, Amy J. Page, Matthew Dowson, Sameh K. ElBadry, Francis M. Barnieh, Robert A. Falconer, Sherif F. El-Khamisy

**Affiliations:** ^1^ School of Biosciences, Healthy Lifespan and Neuroscience Institutes, University of Sheffield, Sheffield, United Kingdom; ^2^ Institute of Cancer Therapeutics, Faculty of Life Sciences, University of Bradford, Bradford, United Kingdom

**Keywords:** aminopeptidase-N, CD13, ATM, cell migration, angiogenesis, DNA repair

## Abstract

Classically, ATM is known for its role in sensing double-strand DNA breaks, and subsequently signaling for their repair. Non-canonical roles of ATM include transcriptional silencing, ferroptosis, autophagy and angiogenesis. Angiogenesis mediated by ATM signaling has been shown to be VEGF-independent via p38 signaling. Independently, p38 signaling has been shown to upregulate metalloproteinase expression, including MMP-2 and MMP-9, though it is unclear if this is linked to ATM. Here, we demonstrate ATM regulates aminopeptidase-N (CD13/APN/ANPEP) at the protein level. Positive correlation was seen between ATM activity and CD13 protein expression using both “wildtype” (WT) and knockout (KO) ataxia telangiectasia (AT) cells through western blotting; with the same effect shown when treating neuroblastoma cancer cell line SH-SY5Y, as well as AT-WT cells, with ATM inhibitor (ATMi; KU55933). However, qPCR along with publically available RNAseq data from Hu et al. (J. Clin. Invest., 2021, 131, e139333), demonstrated no change in mRNA levels of CD13, suggesting that ATM regulates CD13 levels via controlling protein degradation. This is further supported by the observation that incubation with proteasome inhibitors led to restoration of CD13 protein levels in cells treated with ATMi. Migration assays showed ATM and CD13 inhibition impairs migration, with no additional effect observed when combined. This suggests an epistatic effect, and that both proteins may be acting in the same signaling pathway that influences cell migration. This work indicates a novel functional interaction between ATM and CD13, suggesting ATM may negatively regulate the degradation of CD13, and subsequently cell migration.

## 1 Introduction

Ataxia telangiectasia mutated (ATM) signaling plays an essential role in DNA damage repair, as does its fellow PI3K-related protein kinase family member (Savitsky et al., 1995) ataxia-telangiectasia and Rad3 related (ATR) (Blackford and Jackson, 2017). Upon sensing double-strand DNA breaks, ATM recruits the Mre11/Rad50/NBS1 (MRN) complex before phosphorylating substrates including pCHK2, NuMA (Ray et al., 2022; Korovesis et al., 2023) and histone H2AX, signaling for their repair through homologous recombination (HR) or the more error-prone non-homologous end joining (NHEJ). Additional downstream effects can include cell cycle arrest, allowing sufficient time for repair or, should DNA damage be extensive, p53-induced apoptosis (Blackford and Jackson, 2017; Phan and Rezaeian, 2021).

Should the function of ATM be compromised in somatic cells, this can enable the acquisition of tumorigenic mutations, often seen in cancers including breast cancer (Moslemi et al., 2021), neuroblastoma (Southgate et al., 2020) and colorectal cancer (Randon et al., 2019). Germline mutations in ATM can result in the neurological disorder Ataxia-Telangiectasia (A-T), characterized by traits including ataxia, cerebral degeneration and increased cancer risk (Savitsky et al., 1995; Verhagen et al., 2009). Most commonly, truncated transcripts and aberrant splicing are the cause of A-T, with over 300 different mutations being shown to cause this condition (Broekss et al., 2000).

Along with signaling for repair in response to double-strand DNA breaks, ATM also hinders chromatin decompression, hence silencing transcription. Shanbhag et al. (2010) highlight that this could be important for maintaining proximity of break ends following DNA breakage (Harding et al., 2015), with a secondary consequence of transcriptional silencing. ATM-induced transcriptional silencing correlates with H2A ubiquitylation, regulated through E3 ubiquitin ligases RNF8 and RNF168; deubiquitylation through USP16 then overcomes the silencing once DNA repair has taken place through 53BP1 and RAD51 recruitment to ubiquitinated H2AX, and hence the site of repair (Messick and Greenberg, 2009; Shanbhag et al., 2010; Fradet-Turcotte et al., 2013; Harding et al., 2015; Walker et al., 2017).

In cancer, ROS accumulation is promoted by the rapid proliferation of cancer cells and the subsequent genomic instability caused (Perillo et al., 2020). Accumulation of ROS triggers the activation of ATM via p38a, negatively regulating ROS levels. P38 then signals for cell migration. Hence, inactivation of ATM, through inhibition or otherwise, results in excessive ROS and subsequent oxidative stress, hindering cell growth. Okuno et al. (2012) identified this a VEGF-independent angiogenic mechanism, with solely a pathological role, such as in cancer progression (Lee et al., 2021).

In the DNA damage response, p38 is activated by ATM (Thornton et al., 2016), showing a cyclical signaling network interconnecting DNA repair and angiogenesis. It has previously been shown p38 upregulates metalloproteinases MMP2 and MMP9 following IL-1beta activation (Huang et al., 2014), however this has not been associated with ATM activity directly in the literature, to our knowledge.

Inhibition of this non-canonical angiogenesis mechanism through ATM inhibition showed to impair angiogenesis, and in combination with VEGF inhibition showed synergistic effect. However, the myriad of side effects known to be associated with ATM inhibitors in cancer therapy cannot be ignored, which are caused by the fundamental role of ATM in DNA damage repair signaling. This was identified by the authors, with the proposal of an ATM inhibitor being developed specifically targeting the angiogenesis activity of ATM (Okuno et al., 2012).

Likewise, H2AX, a DNA damage signaling protein of similar importance, has also been implicated in angiogenesis as an ATR-specific hypoxia response. When hypoxia evokes the phosphorylation of H2AX, endothelial cell proliferation was promoted, with H2AX-deficiency impairing this (Economopoulou et al., 2009).

CD13 is a moonlighting protein with endopeptidase activity, most prominently researched for its role in angiogenesis, with the function of CD13 differing in cancer to normal tissues (Barnieh et al., 2021). This was recently further elucidated by Barnieh et al. (2023), who showed there to be unique, tissue-specific glycoforms of CD13, providing rationale for its tissue-specific function. The enzymatic activity of CD13 is responsible for the degradation of type IV collagen, enabling cancer cell migration (Fujii et al., 1995). Angiogenic signaling has been shown to activate CD13 through Ras signaling via Ets-2 phosphorylation. The CD13 sequence lacks a HIF-1 consensus site (Bhagwat et al., 2001), a classic hypoxia and angiogenesis signaling protein, but possesses Ets family binding sites, with Ets-1 known to be involved in angiogenesis and tumor cell migration. Knockdown of Ets-2 results in CD13 promoter activity being downregulated, impairing capillary network formation. Other downstream targets of CD13 include Erk1/2, MEK1/2 and Src (Petrovic et al., 2003).

The role of both ATM and CD13 in angiogenesis is established, but any functional interaction between the two is yet to be elucidated. Our work highlights that ATM activity regulates CD13 expression and may have subsequent effect on cell migration.

## 2 Materials and methods

### 2.1 Cell culture

Ataxia telangiectasia cell lines, pEBS and pEBS-YZ5 (Ziv et al., 1997), were kindly provided by Yoshi Shiloh (Tel Aviv, Israel), and cultured in Dulbecco’s Modified Eagle Medium Nutrient Mixture (DMEM) supplemented with 10% fetal bovine serum, 100 μg/mL penicillin and streptomycin, 1% L-glutamine. SH-SY5Y cells were cultured in DMEM F-12 + GlutaMAX supplemented with 10% fetal bovine serum, 1% non-essential amino acids, 100 μg/mL penicillin and streptomycin.

### 2.2 Western blotting

**FIGURE 1:**
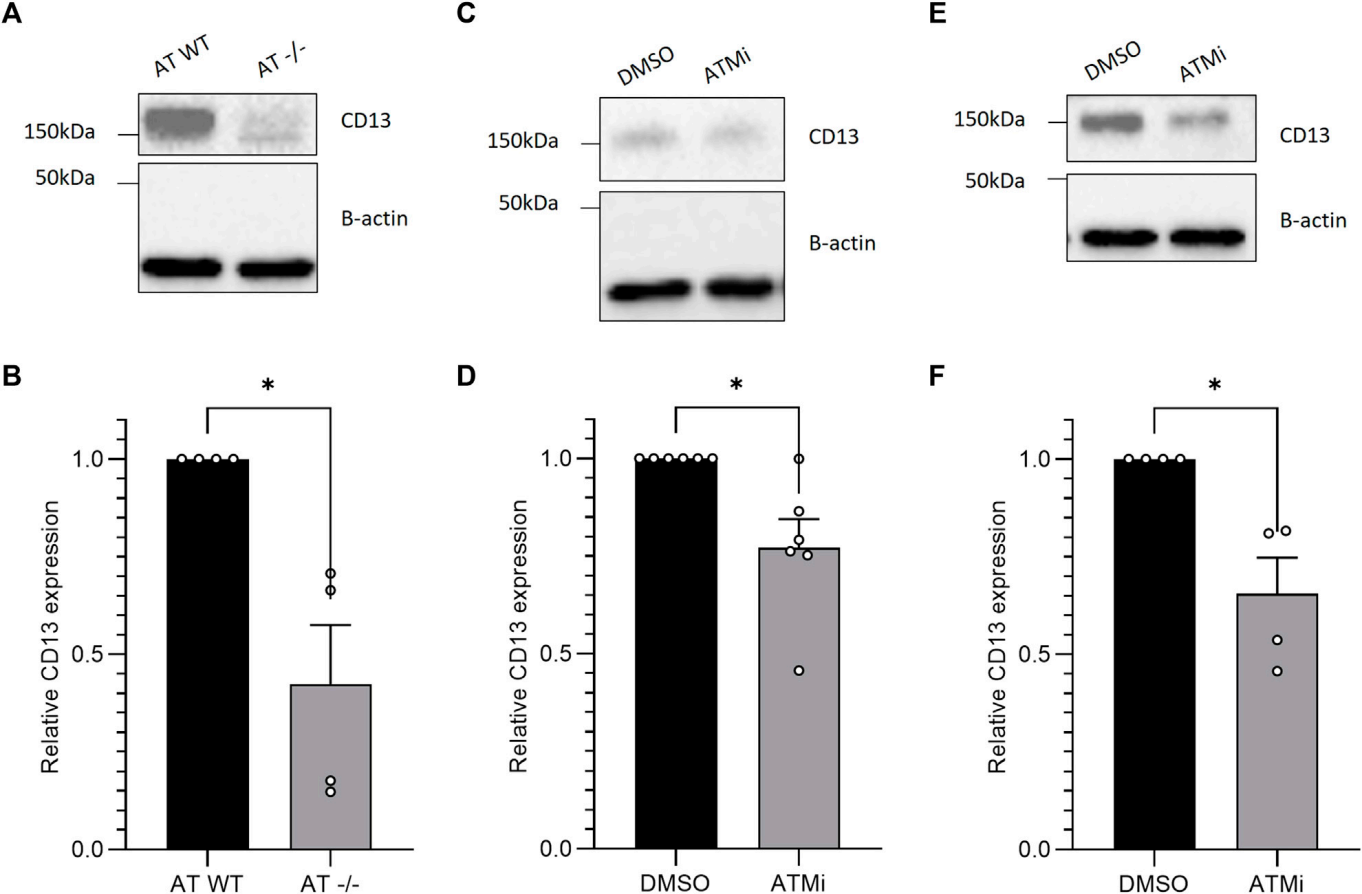
Western blotting of CD13 expression in: **(A)** CD13 expression of AT cell lines pEBS-YZ5 (AT-WT) and ATM knockout pEBS (AT-/-) at baseline, then quantified in **(B)** (*n* = 4); **(C)** AT-WT cell line −/+ 10 μM ATM inhibition (ATMi) treatment for 16 h, quantified in **(D)** (*n* = 4); **(E)** SH-SY5Y cell line −/+ ATM inhibition treatment for 16 h, quantified in **(F)** (*n* = 4). Western blots quantified, SEM error bars. Unpaired *t*-test with Welch’s correction, *p < 0.05.

**FIGURE 2:**
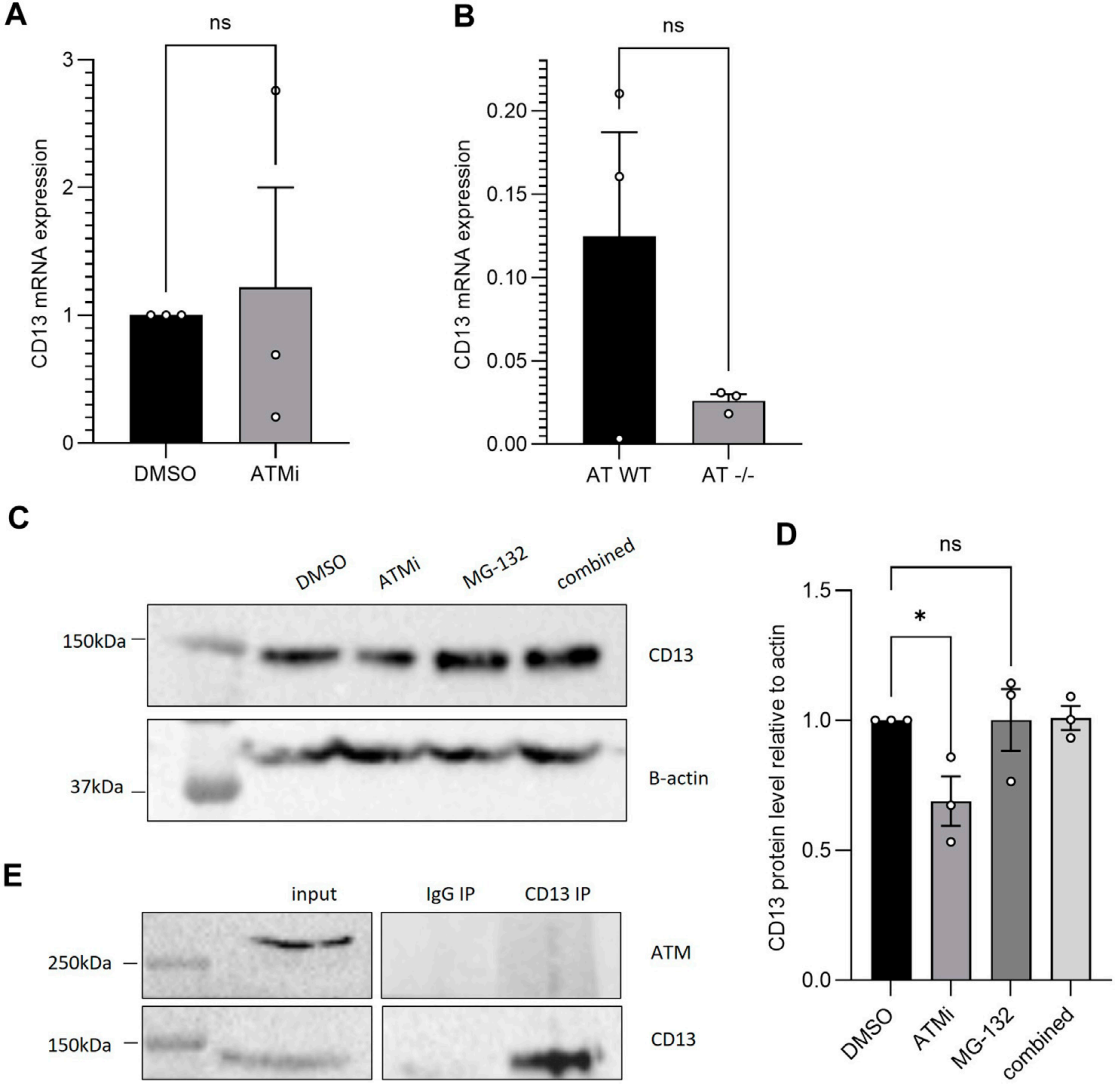
**(A)** qPCR CD13 mRNA expression of SH-SY5Y cells−/+ 10 μM ATM inhibition treatment for 16 h, normalised to B-actin (*n* = 3), SEM error bars, unpaired *t*-test with Welch’s correction; **(B)** qPCR CD13 mRNA expression of AT-WT and AT-/- cell lines, normalised to B-actin (*n* = 3), SEM error bars, unpaired *t*-test. **(C)** CD13 protein expression following 6h treatment with 10 μM ATMi and/or proteasome inhibitor MG-132 in AT-WT cells **(D)** which was then quantified and analysed with one-way ANOVA (*n* = 3), *p < 0.05. **(E)** CD13 co-immunoprecipitation in SH-SY5Y cells, blotting for ATM. No interaction detected.

Cells were lysed with SDS loading buffer and boiled at 95°C for 5 min, then samples were run on a homemade gradient gel (4%–20%) for SDS-PAGE. Protein was transferred by semi-dry transfer. Following transfer, the membrane was blocked using 5% milk in 1 X TBST. Membrane was incubated overnight with primary antibody in 5% milk in 1X TBST, rotated at 4°C. CD13 antibody used 1:100 (3D8 sc-13536, Santa-Cruz); B-actin used 1:1000 (#ab8226, Abcam); ATM antibody used 1:1000 (#ab32420, Abcam). Following 1X TBST washes, membrane was incubated in goat anti-mouse secondary antibody (Bio-Rad, United States) at room temperature for 1 h. Membrane was washed with 1X TBST, then Clarity Western ECL solution was applied before imaging with Chemidoc (Bio-Rad, United States). Bands were quantified using Image Lab software’s band finder tool, based on adjusted volume values. Statistical testing was performed, using unpaired *t*-test for Figures 1, 2A,B, and one-way ANOVA for Figures 2D, 4.

### 2.3 qPCR

SH-SY5Y cells at approximately 70% confluency were treated with 10 µM ATM inhibitor (KU55933; SML1109, Sigma Aldrich) or DMSO for 16 h. Cells were lysed and homogenized using the RNeasy Mini Kit (74136, Qiagen), then RNA was extracted using the RNeasy Mini Kit as per manufacturer’s instructions. Then, cDNA was synthesized using the High Capacity cDNA Reverse Transcription Kit (Applied Biosystems). Samples were then diluted 10-fold and QuantiNova SYBR Green PCR Kit (7500) (208057, Qiagen) used with 700 nM primers. Primer sequences were: GAC​GCT​GAG​ACC​GTA​CCT​C (forward primer), TCA​GTC​TTG​TCA​ATG​TCG​GGG (reverse primer); primer sequences were acquired through PrimerBank, PrimerBank ID: 157266299c2 (Spandidos et al., 2010). Statistical testing was then performed using unpaired *t*-test with Welch’s correction where normalization was carried out.

### 2.4 Co-immunoprecipitation

CD13 antibody (3D8 sc-13536, Santa-Cruz) or mouse IgG and Protein G Dynabeads (Thermo Fisher), were incubated for 1 h at room temperature, before lysates were added and rotated for 2 h at 4°C. Beads were washed four times with NP-40 buffer, then boiled in 1X SDS loading buffer. IP and input were then run on a 4%–15% homemade protein gel and analysed by western blotting.

### 2.5 Scratch assay (migration assay)

At 90%–100% confluency in a 24 well plate, cells were pretreated with 100 µM CD13 inhibitor, bestatin hydrochloride (B8385, Sigma Aldrich), for 16 h and/or 10 µM ATM inhibitor KU55933 (531978, Sigma Aldrich) for 2 h prior to inflicting a wound with a sterile tip. Then, cells were imaged on a light microscope, and again after 24 h. Distance migrated was measured using Fiji ImageJ software, then normalized to the control condition. Statistical testing was then performed using one-way ANOVA.

### 2.6 RNA-seq data acquisition and analysis

Raw, pre-processed gene counts were taken from Gene Expression Omnibus series GSE161922 (https://www.ncbi.nlm.nih.gov/geo/query/acc.cgi?acc=GSE161922) which originally came from Hu et al. (2021). Count normalization and differential expression of genes, considered *p* < 0.01 after adjusting for multiple comparisons, were obtained using the DEseq2 (Version 1.42.0) Bioconductor package in R (Version 4.1.3). Gene annotation was achieved using the org. Mm.e.g.,.db database from AnnotationDbi (Version 3.18.0). Angiogenesis associated genes were isolated using the gene ontology GO:0001525. GSEA was performed using the clusterProfiler (Version 3.18.1) package in R.

## 3 Results

To explore the effects of ATM on downstream signaling, ataxia telangiectasia cell line pEBS was used as a model, along with its “wildtype” (WT) counterpart pEBS-YZ5, which has the wildtype ATM cDNA reintroduced (Ziv et al., 1997). Throughout this paper, these cell lines shall be referred to as AT-WT (pEBS-YZ5) and AT−/− (pEBS).

Incidental findings revealed by western blotting, at baseline, that AT−/− cells express a significantly lower CD13 level compared to the ATM-expressing line, AT-WT (Figures 1A, B) (*p* < 0.05, *n* = 4). There was less than half the CD13 expression seen in cells lacking ATM expression, compared to those with wild-type ATM, indicating ATM could regulate CD13 expression downstream. VEGF was also probed for with no conclusive difference observed and therefore was not included in this manuscript.

To test if ATM catalytic activity is required for regulating CD13 levels, and to confirm the differing CD13 protein expression was not due to other additional genetic changes between the cell lines, AT-WT cells were then treated with ATM inhibitor (ATMi) KU55933 for 16 h. The downregulation of CD13 upon ATM inhibition was replicated, with a statistically significant ∼30% reduction observed in CD13 expression (Figures 1C, D) (*p* < 0.05, *n* = 4).

To further confirm the effect of ATM activity on CD13 protein expression was legitimate, and not an effect unique to this cell line, neuroblastoma cell line SH-SY5Y was also treated with ATMi for 16 h. SH-SY5Y cells mimicked the effect seen between the AT lines when treated, showing a statistically significant ∼35% reduction in CD13 expression upon ATM inhibition (Figures 1E, F) (*p* < 0.05, *n* = 4). This strongly supports the notion that ATM activity regulates CD13 protein level.

To understand the cause for the changes in CD13 expression, RT-qPCR was performed using SH-SY5Y cells with or without 16 h ATMi treatment. Despite reduced protein level, ATM inhibition did not have a significant effect on mRNA levels (Figure 2A) (*n* = 3), with a large range in CD13 mRNA expression between samples. The same result was observed between AT-WT and AT−/− cells (Figure 2B). This suggests changes in protein level may be due to altered degradation following CD13 translation, for example, rather than via downregulation of transcription of CD13 or degradation of its transcripts. To further understand this, AT-WT cells were treated with proteasome inhibitor, MG-132 (Z-Leu-Leu-Leu-al, C2211, Sigma Aldrich), with and without ATMi, for 6 h. Incubation with MG-132 lead to restoration of CD13 protein levels in cells treated with ATMi (Figures 2C, D). This further supports the theory that ATM is regulating CD13 via the proteasome. To confirm whether this was through a direct interaction, CD13 co-immunoprecipitation was performed, and no detectable interaction was observed between CD13 and ATM (Figure 2E). This makes direct stabilization unlikely, but does not rule out the possibility of a transient interaction that may not be picked up by co-immunoprecipitation. Equally, ATM could be having an indirect effect on degradation through post-translational modifications, for example.

**FIGURE 3:**
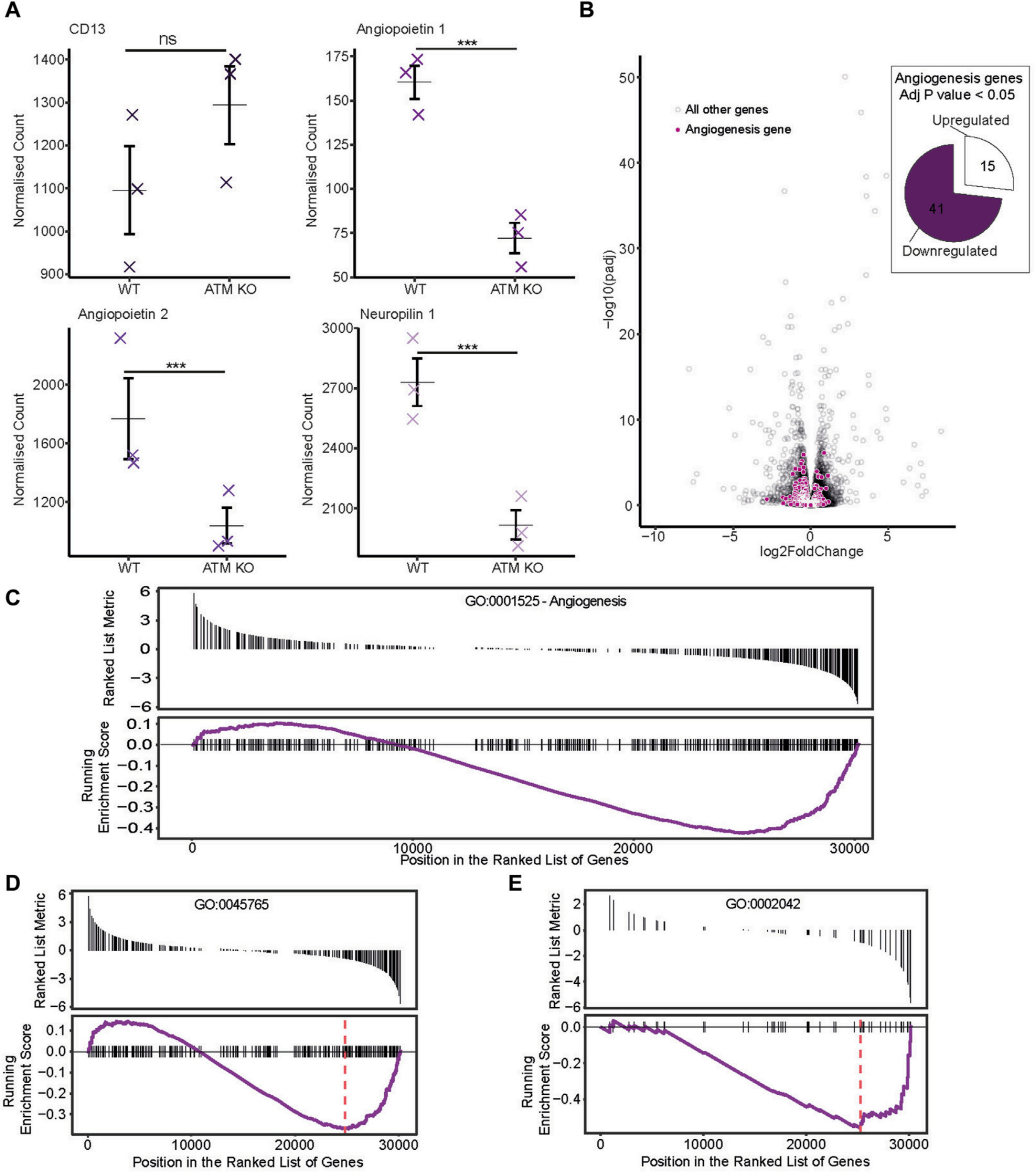
Publicly available RNAseq dataset from Hu et al. (2021) was sourced, whereby mice were injected with ATM proficient or deficient B16F10 melanoma cancer cells. **(A)** Normalized counts of genes potentially relevant to angiogenesis progression. Individual sample counts plotted as crosses with the mean represented by a horizontal bar and error bars showing ±1 standard error. **(B)** Differential expression of angiogenesis associated genes. In the volcano plot, genes corresponding to the Angiogenesis gene ontology term GO:0001525 are highlighted in purple. Those with a *p* score of less than 0.05 are represented as a proportion of genes either up or downregulated in the pie chart. **(C–E)** Gene set enrichment analysis was performed and genes for differing angiogenesis associated GO terms plotted with a ranked list metric showing log2 fold change scores in black and running enrichment score as the purple line. Maximum enrichment score is shown by the red dashed line. **(C)** shows scores for the Angiogenesis GO term GO:0001525. **(D)** For GO:0045765 regulation of angiogenesis and **(E)** GO:0002042 cell migration involved in sprouting angiogenesis.

Exploring this further, a publicly available RNAseq dataset from Hu et al. (2021) was sourced from the Gene Expression Omnibus, whereby mice were injected with B16F10 melanoma cancer cells. These either had WT ATM expression or it was knocked-out by CRISPR-Cas9 technology. Data was reanalyzed to assess CD13 expression and angiogenic pathways. CD13 mRNA level changes here were also insignificant (Figure 3A), supporting the qPCR data. Again, indicating ATM modulates CD13 at the protein rather than transcript level, either directly or indirectly.

It is widely accepted that ATM regulates angiogenesis. To confirm this in the RNAseq dataset, expression of genes involved in the angiogenesis pathway were analyzed. Angiopoietin 1 and 2, and neuropilin 1 all showed significant downregulation upon ATM inhibition (Figure 3A). Globally, angiogenesis pathways were generally downregulated too. With ATM KO, 41/56 differentially expressed angiogenesis genes (*p* < 0.05) were downregulated with only 15/56 upregulated (Figure 3B).

Next, a gene set enrichment analysis was carried out to identify if angiogenesis related pathways were significantly differentially expressed under ATM KO conditions. This again showed the trend of a greater proportion of downregulated genes in the ATM KO line using the angiogenesis gene ontology term GO:0001525 (Figure 3C), though this was not significant (*p* > 0.05). However, using the more specific gene ontologies GO:0045765 (regulation of angiogenesis) and GO:0002042 (cell migration involved in sprouting angiogenesis) we do see a significant downregulation of these pathways in the ATM KO line with Enrichment Score −0.37, *p* < 0.05 and Enrichment Score −0.56, *p* < 0.05 respectively (Figures 3D, E).

**FIGURE 4:**
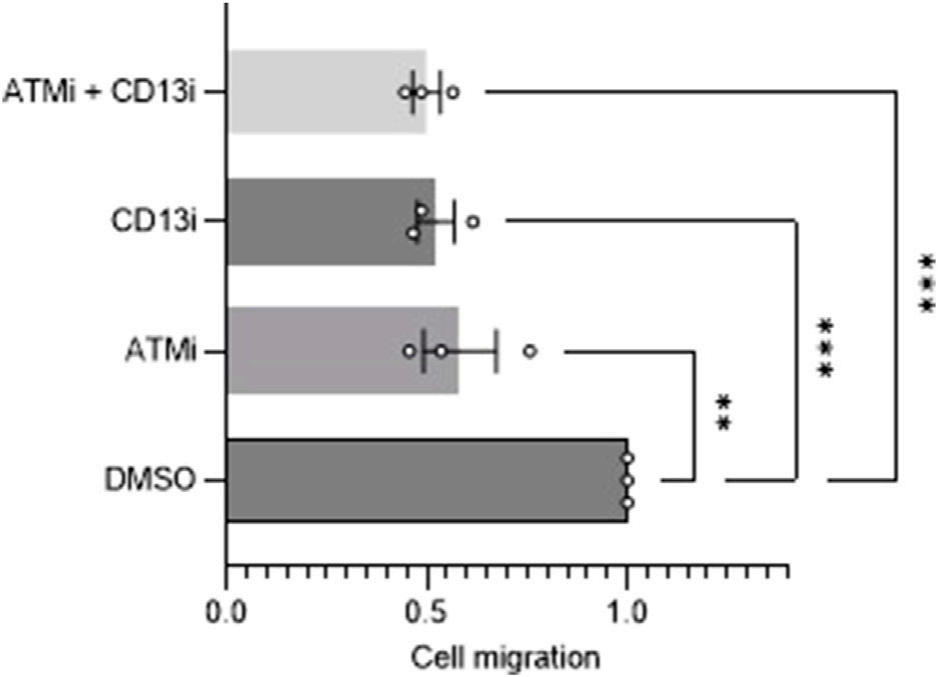
Migration analysis of ATM and CD13 inhibition. Scratch assay analysis of cell migration after 24 h of cells treated with DMSO control, 10 uM ATMi, 100 uM CD13i or ATMi and CD13i in combination (*n* = 3). One-way ANOVA statistical analysis, **p < 0.01, ***p < 0.001. SEM error bars.

CD13 is a known player in angiogenesis and migration, with its role thought to be unique to tumor neoangiogenesis specifically. ATM is also an established regulator of angiogenesis, indicating ATM rationally could modulate angiogenesis via the regulation of CD13. To investigate this, a commonly used migration assay, a wound-healing scratch assay, was performed using SH-SY5Y cells (*n* = 3). ATM inhibition alone caused a ∼40% impairment of migration (*p* < 0.01), and CD13 inhibition did also by ∼50% (*p* < 0.001) (Figure 4). In combination, ATM and CD13 inhibition together caused a 50% (*p* < 0.001) impairment of migration relative to DMSO control. This is not an additive effect, and was not significantly greater than either drug alone, with only a 2% difference in the means of CD13 inhibitor (CD13i) alone and ATMi + CD13i (*p* = 0.997). This suggests an epistatic effect, and that both proteins may be acting on the same signaling pathway that influences cell migration, in addition to their known targets.

## 4 Discussion

The positive progress of ATM inhibitors in clinical trials thus far has primarily been attributed to the disruption of the DNA damage response (Jin and Oh, 2019). However, across the literature multiple roles of ATM have been implicated in cancer. In this preliminary work, we demonstrate a shared signaling pathway between ATM and CD13, subsequently affecting cell migration and inferred impairment of downstream angiogenesis.

Here we showed loss of ATM in ataxia telangiectasia cells occurs with a reduced CD13 expression (Figure 1A). Reduced CD13 expression was also observed when AT-WT and SH-SY5Y cells were treated with an ATM inhibitor (Figures 1C–F). At the mRNA level, however, CD13 levels did not change significantly (Figures 2A, B), suggesting that ATM may modulates CD13 at the protein, not transcriptional, level. This was further supported by the observation that incubation with MG-132 lead to restoration of CD13 protein levels in cells treated with ATMi (Figures 2C, D). However, this is unlikely to be via direct interaction, as co-immunoprecipitation detected no interaction between ATM and CD13 (Figure 2E). To confirm the effect of CD13 was dependent on ATM, and had a downstream effect on angiogenesis, migration assays were performed. Both CD13 and ATM inhibitors alone cause impaired migration, but combined showed no additive effect (Figure 4), suggesting that ATM may modulate CD13 to influence migration within the same pathway. However, this may also be attributed to indirect signaling effects. It is important to note that the CD13 inhibitor used in this study is not entirely selective and inhibits other proteases (Lendeckel et al., 2023).

Ataxia telangiectasia patients, along with more classical traits, have vasculature abnormalities (Meshulam et al., 2012). However, ATM-regulated angiogenesis is understood to be only in pathological conditions such as cancer (Okuno et al., 2012). In contrast, the role of CD13 in neoangiogenesis is not cancer specific. Our work demonstrated ATM inhibition and loss to impair CD13 expression in cancer cell line SH-SY5Y, but also an AT cell line. CD13 is not the only aminopeptidase involved in angiogenesis. Possibilities could be that the role of ATM in angiogenesis via other proteases such as MMP-2/9 is solely in malignant tissues, and that this is not true for CD13-mediated angiogenesis.

Future work in elucidating this pathway would be to understand how ATM regulates the expression of CD13 at the protein level. ATM is known to regulate proteasomal degradation by negatively regulating the ISG15 pathway (Wood et al., 2011). However, how ATM regulates this pathway is unclear. Additionally, ATM is also a known regulator of autophagy (Stagni et al., 2020). We propose that downregulation of CD13 at the protein level is due to negative regulation of protein degradation by ATM through a pathway such as ISG15. We saw that inhibition of ATM with proteasomal inhibition had no significant effect on CD13 expression, supporting the hypothesis that ATM is regulating CD13 via the proteasome.

Knowledge that ATM regulates CD13 is important in the development of novel therapeutic strategies harnessing the role of CD13 in cancer angiogenesis. Within this paper, bestatin was used, a potent aminopeptidase inhibitor. Bestatin has been shown to effectively hinder cancer progression in solid tumor and hematological malignancies in clinical trials (Bauvois and Dauzonne, 2006; Barnieh et al., 2021). Despite this, progress in translation to clinical has haltered. Other approaches to CD13 therapeutics include prodrugs, whereby hydrolysis of a compound by the aminopeptidase leads to activation of compounds focused around CD13 highly expressing tissues. Thus far, the compounds lack tumour-selectivity (Wickström et al., 2011). From our work, going forward, tumour-targeting prodrugs hydrolyzing CD13 should avoid targeting ATM and its downstream targets; not doing so may cause impaired ATM signaling and subsequent reduced CD13 expression, hindering the efficacy of the therapeutic. In contrast, PARP inhibition is known to activate ATM (Aguilar-Quesada et al., 2007). Therefore, PARP inhibitors, or inhibitors of other DNA repair proteins, could be good targets for prodrug development that rely on activation via CD13.

To conclude, we have demonstrated cell migration, and inferred angiogenesis, is modulated by ATM via CD13 signaling. ATM likely impairs the degradation of CD13 at the protein level by negatively regulating proteasomal degradation, hence on inhibition of ATM, CD13 levels decrease, and migration is impaired. This highlights CD13 as a new player in ATM-mediated angiogenic signaling.

## Data Availability

The original contributions presented in the study are included in the article/Supplementary Material, further inquiries can be directed to the corresponding author.
